# The emerging roles of TLR and cGAS signaling in tumorigenesis and progression of ovarian cancer

**DOI:** 10.3389/fphar.2022.1072670

**Published:** 2022-12-16

**Authors:** Zhen Zhang, Hong Zhao, Chu Chu, Xiaoxiao Fu, Yonglin Liu, Li Wang, Ran Wei, Ke Xu, Lihua Li, Xia Li

**Affiliations:** ^1^ Innovative Institute of Chinese Medicine and Pharmacy, Shandong University of Traditional Chinese Medicine, Jinan, Shandong, China; ^2^ School of Clinical and Basic Medicine, Shandong First Medical University and Shandong Academy of Medical Sciences, Jinan, Shandong, China; ^3^ Department of Systems Medicine and Bioengineering, Houston Methodist Cancer Center, Houston, TX, United States; ^4^ Department of Obstetrics and Gynecology, The First Affiliated Hospital of Shandong First Medical University, Shandong Provincial Qianfoshan Hospital, Jinan, Shandong, China

**Keywords:** Toll like receptors, cGAS, inflammation, ovarian cancer, innate immune system

## Abstract

Ovarian cancer is fatal to women and has a high mortality rate. Although on-going efforts are never stopped in identifying diagnostic and intervention strategies, the disease is so far unable to be well managed. The most important reason for this is the complexity of pathogenesis for OC, and therefore, uncovering the essential molecular biomarkers accompanied with OC progression takes the privilege for OC remission. Inflammation has been reported to participate in the initiation and progression of OC. Both microenvironmental and tumor cell intrinsic inflammatory signals contribute to the malignancy of OC. Inflammation responses can be triggered by various kinds of stimulus, including endogenous damages and exogenous pathogens, which are initially recognized and orchestrated by a series of innate immune system related receptors, especially Toll like receptors, and cyclic GMP-AMP synthase. In this review, we will discuss the roles of innate immune system related receptors, including TLRs and cGAS, and responses both intrinsic and exogenetic in the development and treatment of OC.

## 1 Introduction

Inflammation is a complex and crucial process, which must be precisely controlled to efficiently eliminate infections, heal injured tissues, and thus maintain homeostasis (Kotas and Medzhitov, 2015). Inflammation is routinely considered to be an exclusive feature restricted to innate immune responses, which are characterized by rapid onset elicited by a series of innate immune related receptors, mainly including Toll like receptors (TLRs), cyclic GMP-AMP synthase (cGAS), and RIG-I-like receptors (Loo and Gale, 2011; Li and Chen, 2018; Fitzgerald and Kagan, 2020). The receptors are called pattern recognition receptors (PRRs), and triggered by conserved structures of invading bacteria and viruses, known as pathogen associated molecular patterns (PAMPs). Besides, in the healing process for tissues, normal constituents, identified as damage associated molecular patterns (DAMPs), were produced or leaked from dying or wounded cells, could also be recognized by PRRs, and initiate inflammatory responses in the absence of pathogen infections, which were called sterile inflammation (Gong et al., 2020). Both PAMPs and DAMPs mediated inflammatory responses, if not stringently supervised, could occur repeatedly and turn into chronic inflammation and eventually in some cases cause cancer (Gupta et al., 2018).

In order to decipher the roles and mechanisms of inflammation in cancer development, it is fundamental to figure out what induces inflammation before tumor formation. The concept of tumor development and progression has shifted from “cancer cell focus” to “causative microenvironment,” where the surrounding stromal cells, including fibroblast, vascular cells and inflammatory immune cells are taken into account (Yang et al., 2020). It is obvious that the inflammatory condition is not only attributed by immune cells, but also fueled by other stromal cells since the innate immune related receptors are widely expressed in the stromal and certain cancer cells. From this view, any stimulus that are competent for inflammation induction might be correlated with cancer. Indeed, it is speculated that 15%–20% cancer patients suffer from infection, chronic inflammation, or autoimmunity at the same tissue or organ site prior to cancer development (Grivennikov et al., 2010). Although the rest 80%–85% cancer does not precede from long-existing chronic inflammation, cancer cells are examined to be able to recruit immune cells and leverage inflammatory mediators to re-program the tumor microenvironment (TME) to facilitate tumor progression.

Apart from PRR mediated inflammation participating in cancer development or metastasis, there is another important type of inflammation which is evoked in the course of anti-tumor therapies, including chemo-, radio-, or immunotherapies (Greten and Grivennikov, 2019). Generally, this type of inflammation is beneficial for cancer treatment in the aspect that DAMPs and neo-antigens released by necrotic cancer cells can promote the production of immune-stimulatory cytokines and anti-tumor T cell response (Hernandez et al., 2016). However, the ultimate outcome is largely orchestrated by a series of PRRs on cancer cells and stromal cells, which their activations could turn the active state of cancer-immunity to the suppressive one in the course of treatment that leads to therapeutic resistance (Gajewski et al., 2006). As a result, the profound mechanism of therapy related inflammation attracted intensive attentions with the aims to better harness TLR, RLR, or cGAS mediated inflammatory responses in combination with the standard treatments to accelerate cancer regression and maximize patient response.

Ovarian cancer (OC) with a leading death rate of gynecological cancers has been intensively investigated in its development, metastasis, chemo-resistance, and novel strategies for immunotherapies (Savant et al., 2018). OC could be classified into three categories in view of its histological originations, including surface epithelium tumor, germ cell tumor, and stromal cell tumor, among which epithelial ovarian cancer (EOC) accounts for 85%–90% of all OC cases. At the early stages, the cancer is confined to the ovaries (stage I), or with limited spread in the pelvic region (stage II) for EOC patients. At the late stages (III–IV), EOC is often accompanied with distal metastasis outside of the abdomen, including the liver, the fluid in the lungs, the spleen, and even the brain (Lheureux et al., 2019). When being treated timely before tumor spread, the 5-year survival rate for EOC is as high as 90%. However, over 75% of EOC patients were diagnosed at late stages with distal metastasis, and the 5-year survival rate will drop to 30%, which makes EOC quite lethal for the patients. At present, the standard therapeutic strategy is surgical debulking followed by several rounds of chemotherapy based on platinum or taxane (Poveda et al., 2014). However, patients with OC often develop chemoresistance and strategies are also needed to reduce the side effects of extensive chemo treatment. Increasing evidences have clarified that inflammation participates in each process of tumorigenesis, malignant transformation and clinical treatment. A series of literatures have revised the relationship between inflammation and tumor promotion and chemo-resistance in OC from various aspects. For examples, Zhu et al. (2018) concluded that inflammatory variate of neutrophil-lymphocyte ratio (NLR) and platelet-lymphocyte ratio (PLR) were negatively associated with OC patients’ survival, which means the higher inflammatory conditions, the poorer survival rates for OC patients; Savant et al. (2018) proposed that targeting of inflammatory mediators, especially IL-6 and TNF-α is prospective in treating OC patients; Mor et al. (2011) summarized the link between TLR mediated inflammation and cancer stem cell renewal and pointed a promising strategy to reduce OC recurrent rate by TLR intervention; and Liu et al. (2022) confirmed that inflammasome activation played key roles in inducing chemo-resistance, and put forward that targeting of molecular components involved in inflammasome activation might contribute to alleviate OC progression. Based upon these previous knowledge, in this review we primarily focus on PRRs, including TLRs, and cGAS in the OC progression. The literatures were retrieved by searching “(Toll like receptor) and (ovarian cancer)” or “[(cyclic AMP-GMP synthase) or (stimulator of interferon response cGAMP interactor 1)] and· (ovarian cancer)” respectively, and are carefully screened and included with fundamental research data to draw a conclusion. We discussed the expression patterns of the receptors in OC tumors and TME immune cells, and their differential contributions to tumor progression and chemo-resistance in the aspects of inducing inflammatory conditions, and modulating immune capacity of OC tumors, with the aim to prospect precise and combinational targeting PRR for treatment of OC with diverse aggressive properties.

## 2 TLRs and ovarian cancer

### 2.1 Overview of TLR signaling pathways

TLRs are the first gene family identified as PRRs, the function of which has been mostly well characterized for the recognition of a series types of PAMPs (Fitzgerald and Kagan, 2020). The receptor family members belong to type I transmembrane proteins, and structurally they are composed of an extracellular leucine-rich repeats (LRRs), a transmembrane domain, and a cytosolic Toll-IL-1 receptor (TIR) domain. LRRs mediate the recognition of PAMPs, and the intracellular domain facilitates the downstream signaling transduction (Gay and Gangloff, 2008). In mammalian, there are more than 12 TLR members, which are capable of recognizing PAMPs derived from all kinds of infection, including parasites, fungi, bacteria, mycobacteria, and viruses (Kawai and Akira, 2011). The recognition has been closely related to the sub-cellular localization of TLRs. For examples, cell surface-localized members TLR2, TLR4, and TLR5 could recognize lipoproteins, lipopolysaccharide, and flagellin respectively. While, for the cytosolic-localized TLRs, TLR3 directly binds double stranded RNA (dsRNA), TLR9 senses DNA, TLR7 and TLR8 are assigned for single stranded RNA (ssRNA) discrimination (Kawai and Akira, 2011). Apart from pathogen derived PAMPs, DAMPs are important TLRs ligands for inflammation induction. For examples, DAMP related HMGB1 could be detected by TLR2, TLR4, and TLR9; TLR3, TLR7, and TLR9 could recognize mRNA, microRNA, and mitochondrial DNA respectively (Gong et al., 2020). In addition, TLR2 and TLR4 shared a large portion of identical DAMP ligands, including biglycan, decorin, histone, heat shock proteins (HSPs), which are released by damaged or dying cells. Besides, paclitaxel, a frequently administered chemotherapeutic reagent, has also been identified as a TLR4 ligand (Byrd-Leifer et al., 2001).

Upon TLR binding with ligands, the adaptor proteins will be subsequently recruited, especially MyD88 or TRIF, to activate the downstream signaling pathways, which will finally boost the yield of cytokines, chemokines, and type I interferons (Premkumar et al., 2010). Moreover, the production will lead to the infiltration of neutrophils, differentiation or motivation of macrophages, and maturation of dendritic cells, which jointly help to eliminate pathogens and induce adaptive immunity restricted to the infected sites (Dajon et al., 2017). The process of inflammation induction by TLRs is in parallel with the evolvement of TME, which collectively directs the state of tumor progression or remission.

### 2.2 TLRs expression in ovarian tissue and OC cancer cells

TLRs are found in multiple tumor types, and their expressions in ovarian tissue have been clarified. TLRs expression was initially screened in ovarian tissues that TLR2, TLR3, TLR4, and TLR5 are basically expressed with high levels on the surface epithelium of normal ovaries (Zhou et al., 2009). These TLRs are also abundantly expressed in many OC cell lines. Further studies by the same group identified that TLR6 and TLR8 are differentially expressed in benign and malignant OCs, while TLR1, TLR7, and TLR9 are marginally expressed in ovarian tissue. Many studies have highlighted that TLRs activation in OC tumor cells are closely related to cancer aggressiveness, resistance of treatment, adverse clinical outcome, and newest progress in this filed is reviewed in the following sections.

### 2.3 TLR4 contributes to chemo-resistance and is targetable for OC

TLR4 signaling activation requires an accessory partner, named myeloid differentiation protein-2 (MD-2), which is responsible for recognition of TLR4 ligands, and helps to deliver the ligands by forming heterodimer with the receptor (Kim et al., 2007). Ligand bound TLR4 dimerizes on the membrane, recruits MyD88, and results in early activation of NF-κB. Besides, a portion of dimerized TLR4 translocate to the endosome, recruit TRIF, and lead to late phase NF-κB activation and interferon regulatory factor 3 (IRF3) independent of MyD88 (Zanoni et al., 2011). Both pathways, especially TLR4/MyD88, have been elucidated for the role in OC progression and chemo-resistance.

TLR4 is frequently expressed in OC tissues. Presence of TLR4-immunoreactivity was spotted in both cytoplasm and surface of OC tumor cells in paraffin-embedded OC patient tissue sections, and the same expressional pattern for TLR4 was also founded in A2780, CP70, R182, and R179 OC cell lines (Kelly et al., 2006). MyD88, the adaptor protein of TLR4, was distinctively expressed in EOC but not observed in normal ovarian tissue (Zhu et al., 2012) suggesting the tumorigenic role of the TLR4-MyD88 signaling in OC. The clinical significances for TLR4 and MyD88 expression in ovarian tissue have been validated, and it is revealed by Li et al. that both elevated expression of TLR4 and MyD88 predicted poorer overall survival in EOC patients. Furthermore, high expression of TLR4, MyD88, and activated NF-κB signaling were significantly associated with DAMPs, such as HSP60, HSP70, and HMGB1, suggesting endogenous ligands mediated TLR4/MyD88/NF-kB activation contributes to OC inflammation and aggressive phenotype (Li et al., 2016). In addition, high MyD88 expression has been independently investigated to imply an enhanced metastatic property of EOC (Zhu et al., 2012). Altogether, these findings pointed that hyperactivation of MyD88 dependent TLR4 signaling pathway in ovarian tissue are closely related to malignant progress.

Jiang et al. (2018) tested TLR4 expression in EOC, and found it is increased in advanced EOC patients compared with benign ovarian cysts. Besides, Luo et al. (2015) showed that TLR4 expression level was positively correlated with clinical stage or pathological grade of OC patients, which highlighted the potential for the protein in the regulation of the tumor cell malignant progression. In addition, evidences showed that LPS induced activation of TLR4 is sufficient to promote the proliferation and invasion of SKOV3 cells (Kashani et al., 2020). Kelly et al. further reported the differential effects of LPS induced OC inflammation, growth, and chemo-resistance in MyD88 positive (MyD88^+^) and negative (MyD88^-^) EOC cells through TLR4. They found MyD88 is indispensable for LPS induced OC cell growth. NF-κB activation and induction of cytokines by LPS were only observed in MyD88^+^ cells. Besides, MyD88 induction was also revealed to reverse chemosensitivity of paclitaxel, by specifically protecting the EOC cells from paclitaxel induced apoptosis (Kelly et al., 2006).

TLR4 upregulation was also examined on the surface of 2008C13 OC cells by a series of anti-cancer drugs, including paclitaxel and cisplatin, two most commonly used chemotherapeutic drugs for OC patients (Kashani et al., 2019). Wang et al. (2009) found that knockdown of TLR4 restored the inhibitory effects of paclitaxel on cell growth and impeding cell cycle progression mainly *via* downregulation of XIAP and pAKT in SKOV3 cells. It has also been reported by Szajnik et al. that, in the presence of LPS or paclitaxel, TLR4 was activated to induce cJun phosphorylation and NF-κB activation, which subsequently enhanced production of vascular endothelial growth factor (VEGF), and inflammatory cytokines, such as interleukin (IL)-8, IL-6, and elicited drug-resistance in MyD88^+^ SKOV3 cells, but not MyD88^−^ A2780 cells. TLR4 silencing in MyD88^+^ SKOV3 cells reduced phosphorylation level of cJun, and subsequently deprived paclitaxel resistance. Therefore, MyD88 dependent TLR4 signaling pathway sustains OC progression and chemo-resistance (Szajnik et al., 2009).

**FIGURE 1:**
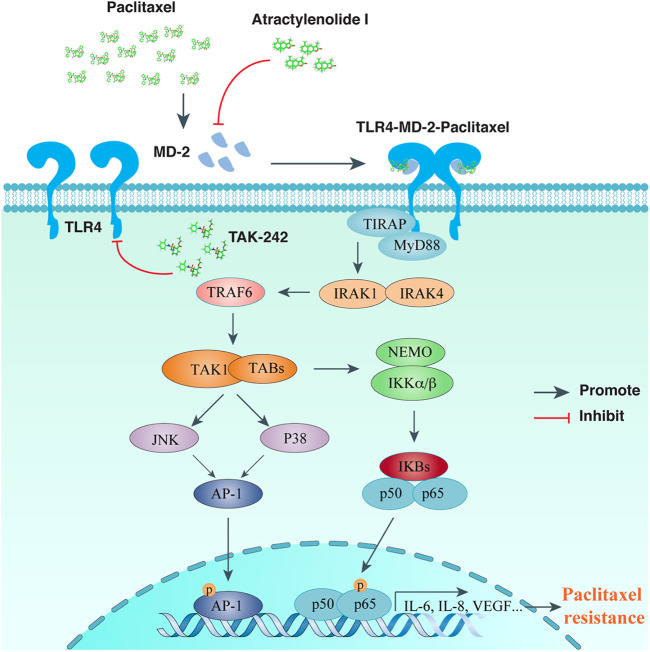
TLR signaling intervention in reversing paclitaxel resistance of ovarian cancer cells. Paclitaxel binds to MD-2, activates TLR4-MyD88 mediated signaling transduction, and leads to chemo-resistance of OCs. Atractylenolide I competes with paclitaxel for MD-2 binding, while TAK-242 inhibits recruitment of TLR4 downstream adaptors, both of which could alleviate paclitaxel mediated chemo-resistance for OCs.

Since TLR4/MyD88/NF-κB signaling pathway has been validated for its role in chemo-resistance, researchers are devoted to investigate the therapeutic potency of various TLR4 inhibitors for the relief of chemo-resistance. Till now, there are several TLR4 inhibitors reported to be functional in alleviating paclitaxel or cisplatin resistance of OC cells, including AO-1, TAK-242, and P-MAPA. In Figure 1, we illustrated the TLR4 signaling pathway and showed the intervening targets of AO-1 and TAK-242 to ameliorate paclitaxel resistance of OC cells.

AO-1, abbreviated from Atractylenolide I, is a sesquiterpene lactone naturally extract from Atracylodes macrocephala Koidz, and possesses immuno-regulatory effects by antagonizing TLR4 activity (Ji et al., 2014). Computational simulation found a docking site of AO-1 with MD-2 similar to TLR4 ligands, including LPS and paclitaxel. In addition to reducing the expression of TLR4 and MD-2, AO-1 was found to reverse chemo-resistance to paclitaxel in OC cells by blocking MD-2 mediated TLR4/MyD88 activation and inflammatory cytokine production (Huang et al., 2014). Besides, the same group also uncovered that AO-1 treated SKOV3 cells ameliorated immuno-suppressive properties, and the cell culture conditioned medium reduced the ability to induce regulatory T (Treg) cells, while enhancing the proliferation and cytotoxicity of T lymphocytes (Liu et al., 2016). Altogether, for tumors expressing TLR4/MD-2/MyD88 in EOC patients, TLR4/MD-2 complex is an appealing target for combined intervention to develop effective immunotherapy strategies of EOC.

TAK-242, formerly reported as an anti-inflammatory agent, has been proved to bind the intracellular domain of TLR4, thereby competing its interaction with the downstream adaptors (Matsunaga et al., 2011). Co-treatment of TAK-242 with paclitaxel, cisplatin, and doxorubicin could dramatically increase the inhibitory effects for these drugs in OC cell proliferation (Kashani et al., 2019; Kashani et al., 2020). In addition, TAK-242 blockade of TLR4 has been discovered to suppress OC cell invasion (Zandi et al., 2019).

In an *in vivo* model of rat with OC induction, Luiz et al. found that the protein aggregate magnesium-ammonium phospholinoleate palmitoleate anhydride (P-MAPA) in combination with cisplatin could raise the efficacy of cisplatin in the aspect of animals’ survival rate, through up-regulating TLR4 and preferentially IFN-γ activation (de Almeida Chuffa et al., 2018). They also found that when used with interleukine 12 (IL12), p-MAPA significantly increased the abundance of CD4 positive (CD4^+^) and CD8 positive (CD8^+^) effector T cells induced by IL12 to enhance the antitumor capacity and point a novel strategy for OC therapy (Silveira et al., 2020).

Uncovering and targeting TLR4 downstream effectors is also a strategy for OC interfering therapy. ABCB1 (P-glycoprotein) and TLR4 expression are simultaneously increased in SKOV3 cells resistant to taxol. Upon TLR4 inhibition, ABCB1 expression was significantly down-regulated, and the cytotoxic activity of taxol was greatly enhanced. On the contrary, ectopic over-expression of ABCB1 blunted the OC cells’ response to taxol. Huang et al. performed high-throughput transcriptomic analysis and identified androgen receptor (AR) as a taxol resistance associated gene in OC by activating TLR4 (Huang et al., 2020). These findings provide direct evidence that the TLR4/NF-κB induced functional genes might act as novel targets to prevent taxol or other kinds of chemo-resistance (Sun et al., 2018).

### 2.4 TLR2 facilitates OC stem cell renewal and TLR9 is involved in chemo-resistance

Although TLR2 has been found expressed on the EOC cells, its diagnostic significance in EOC has been just recently clarified by Małgorzata Sobstyl et al., who found TLR2 was elevated frequently in advanced OC patients (Sobstyl et al., 2021).

Ilana Chefetz et al. reported that TLR2 expressed on OC stem cells and facilitated their self-renewal, which favors tumor recurrence after surgery. The authors have firstly defined a subset of OC stem cells characterized by the expression of CD44 and MyD88 (CD44^+^/Myd88^+^), which is competent to rebuild tumors in mouse model, and maintain a pro-inflammatory microenvironment. They found TLR2 expression was elevated in wounded tumor cells, and by creating an *in vitro* assay of wound healing, peptidoglycan (PGN), a TLR2 agonist, was revealed to accelerate the wound repair, while the dominant negative forms of TLR2 or MyD88 could significantly inhibit the process. Mechanistically, it was fulfilled by a pro-inflammatory condition elicited by activation of TLR2-MyD88-NF-κB pathway (Chefetz et al., 2013). In addition, Cai et al. (2019) reported that TLR9 intervention together with formyl peptide receptor (FPR) targeting reversed chemoresistance and efficiently sensitized OC cells to cisplatin treatment.

### 2.5 TLRs and TME inflammation of OC

There has been little improvement in survival for advanced OC patients over the last few decades, despite significant advances in cancer cell biology. Development of invasive OC is not only attributed to tumor cells escaping from protective immunity, but also the microenvironmental inflammatory condition which abrogates the active immune pressure on cancer progression. The immunosuppressive microenvironment of tumors must be well understood, so that the prospective therapeutic strategies could be conceived. As we begin to comprehend the prohibitory mechanisms, it is revealed that the cancer developmental programs are always orchestrated by the accumulation a series inhibitory immune cells in TME, including regulatory T lymphocytes, tumor associated macrophages (TAMs) with M2 phenotype, neutrophils, myeloid-derived suppressive cells (MDSCs), natural killer (NK) cells, monocytes, dendritic cells (DCs), mast cells, and so on (Jarosz-Biej et al., 2019).

**FIGURE 2:**
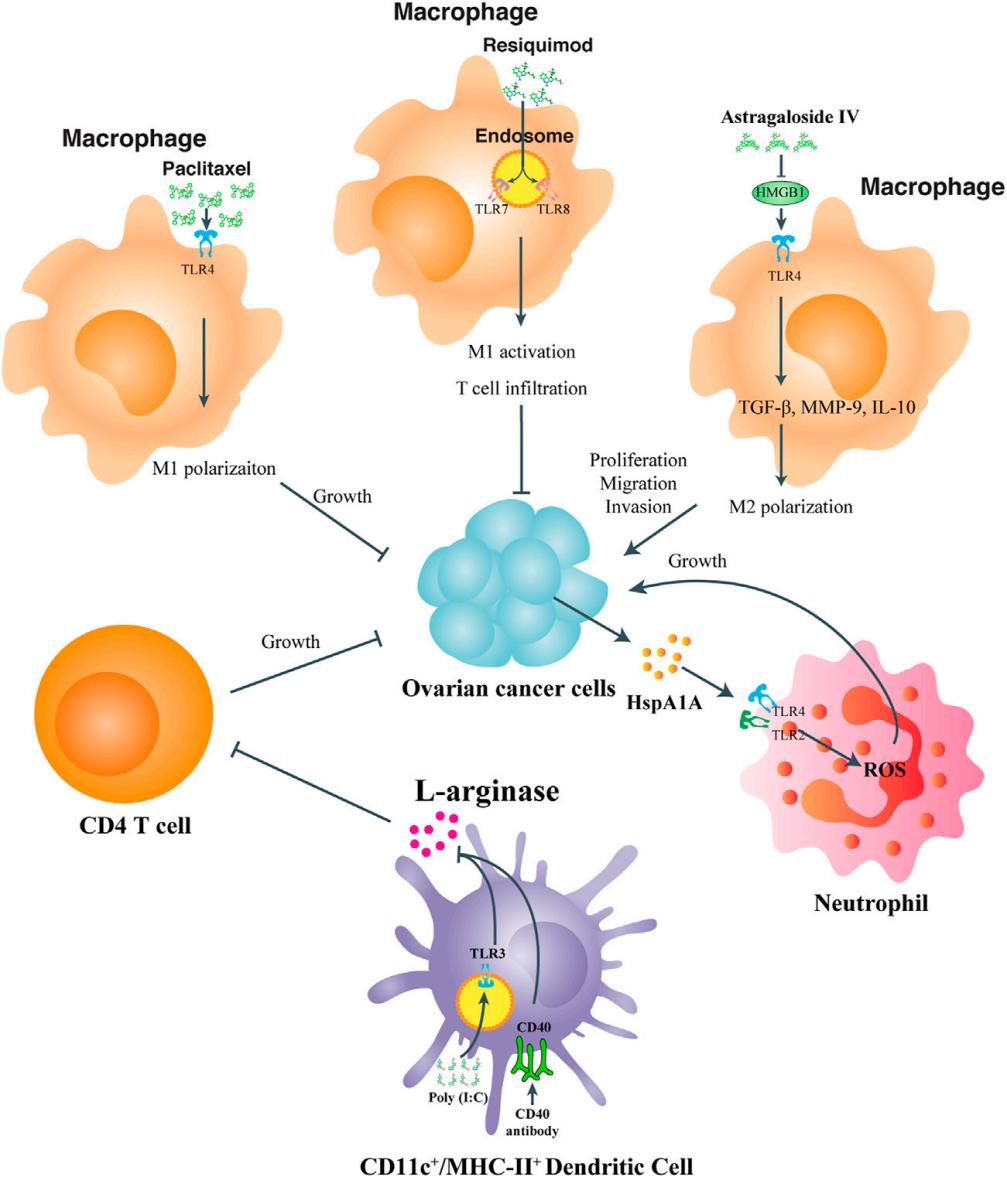
TLR participation in modulating the immune status of tumor microenvironment immune cells. For TME macrophages, paclitaxel, and resiquimod acts on TLR4 and TLR7, 8 respectively to enhance M1 polarization or even T cell infiltration to inhibit OC proliferation or metastasis, while Astragaloside IV targets HMGB1-TLR4 signaling to increase M2 polarization and OC progression. For TME neutrophils, tumor derived HspA1A activates TLR2, and TLR4 signaling transduction to boost ROS production and OC growth. For TME DCs, co-stimulation of TLR3 and CD40 inhibits the activity of L-arginase and relieve its inhibitory effects on CD4 T cells to promote OC remission.

TLR expression, especially in macrophages and DCs is well established during the occurrence of inflammatory response, which subsequently activates numerous transcriptional factors, cytokines and chemokines upon PAMP or DAMP stimulation. A great deal of research has focused on how to dominate TLR signaling to reverse the inflammatory situation which is in favor of OC progression. In Figure 2, we illustrated the effects and mechanism of tumor infiltrating immune cells on OC progression, and summarized the compounds that might act on TLR signaling to positively or negatively affect OC progression.

TAMs are the macrophages infiltrate into the tumor stroma from blood (An and Yang, 2021). In OCs, TAMs are present in both cancer tissue and ascites, which can be cultured by the tumor microenvironment and polarized from M1 into M2 phenotype, facilitating tumor suppressive activities (Rodriguez-Garcia et al., 2021). TLRs related to TAM polarization and re-polarizing TAMs by TLR signaling intervention could be prospective in re-programming the tumor microenvironment. Kang et al. (2021) demonstrated an efficient targeting of TAMs by resiquimod, a TLR7 and TLR8 agonist, through large, anionic liposomes, to repolarize macrophages, promote T cell infiltration, and lower the abundance of Tregs in the tumor microenvironment. Wang et al. uncovered a natural extract, astragaloside IV, from herbal *Radix astragali* inhibited IL4/IL13 induced M2 polarization of THP1 cells by significantly suppressing transcripts of M2 markers. They also uncovered that the medicine attenuates HMGB1/TLR4 transduced signal in M2 like macrophage co-cultured OC cells. More than that, HMGB1 addition to the cultural medium inhibited suppressive ability of astragaloside IV against M2 macrophage and its functional properties (Wang et al., 2021). It was also revealed that the anti-tumor effect of paclitaxel was not only fulfilled by its cytotoxic property, but also achieved by its ability to transform M2 like macrophages to the M1-polarized phenotype dependent on TLR4. Wanderley et al. reported that paclitaxel treatment of patients with OC led to accumulative activation of genes with M1 phenotype prone properties, while the anti-tumor effect of paclitaxel was attenuated in mice with TLR4 conditional knockout on macrophages. They also uncovered that paclitaxel reprogramed M2-macrophages toward an M1-antitumor profile dependent on TLR4 signaling activation (Wanderley et al., 2018). TLR engagement has also been reported in TME cell communication, as Bellora et al. (2014) reported LPS treatment of TAMs boosted the activation of freshly isolated NK cells.

Treg cells (Tregs) are always enriched in the tumor site of OCs (Tanaka and Sakaguchi, 2017). Tregs are important factors in the formation of immunosuppression, while a large portion of Treg infiltration in tumor microenvironment leads to poor clinical outcomes, especially in condition of hindered infiltrating of CD8^+^ cytotoxic T cells (Curiel et al., 2004). Overcoming immunosuppression in the tumor microenvironment is the main obstacle of effectiveness tumor treatment and regulation (Zou, 2005; Shevach, 2009). Xu et al. found OC cells have certain impacts on function of CD4^+^ Tregs through the glucose metabolism pathway, which can be reversed by the regulation of TLR8 activation. Specifically, they discovered that patient derived CD4^+^ Tregs exhibited higher expression of glucose metabolism related genes, and in an *in vitro* co-culture system with SKOV3 cells, Tregs underwent the same change. Activating TLR8 signal by ssRNA40 in the CD4^+^ Tregs led to the accelerated proliferation of naïve T cells by downregulating mTOR and decreasing the relevant glucose metabolic pathways. This study suggested that TLR8 ligand might serve as a potential adjuvant for immunotherapy to reverse the inhibitory function of CD4^+^ Treg cells in OC patients (Shang et al., 2020).

In the OC tissue microenvironment, DCs are the most prevalent type of leukocyte and responsible for initiating and activating T cell dependent tumor immunity. Mechanistically, within TME, DCs adopt, process and deliver the tumor-associated antigens to MHCI/II molecules, and subsequently activate T cells (Zhang et al., 2020a). However, the function of DCs in TME is always reprogramed to perform inhibitory effects on T cell immunity. Scarlett et al. reported that OC TME derived CD11c^+^MHC-II^+^ DCs suppressed T cell function, which could be reversed by co-stimulation of CD40 and TLR3 to an immune-stimulatory status. When being treated for simultaneous CD40 and TLR3 agonizing, the sorted peritoneal DCs from OC-bearing mice showed a significant decrease in l-arginase activity, elevated productions of type I IFN and IL12, and an enhanced capability of antigen processing in *in vitro* and *in vivo*. In addition, co-activation of CD40/TLR3 can induce the activated DCs to migrate to lymphatic sites and improve their ability to present antigens. Accordingly, in the absence of exogenous antigens, the combination of CD40 and TLR3 agonists can enhance T cell-mediated anti-tumor immunity and induce regression of OC (Scarlett et al., 2009).

Monocytes or neutrophils in the TME are also found reprogrammable *via* TLR signaling regulation. Adams et al. reported the antigen-presenting ability of monocyte isolated from OC ascites could be restored by simultaneous activation of TLR4 and TLR9 signaling pathways, in combination with administration of a blocking antibody to interleukin-10 receptor (IL-10R) *in vivo*. Klink et al. (2012) announced that the OC surface protein HspA1A could facilitate cell-cell contact *via* TLR2 and TLR4 on neutrophils, thereby enhancing the inflammatory cytokine production of neutrophils and benefiting tumor progression.

Although emerging evidences have pointed that the TME plays critical roles in OC development and efficacy for immunotherapies, the heterogeneity of OC, including the TME, limited our cognition of OC patient treatment based on most of the *in vitro* data. A recent research takes use of the Cancer Genome Atlas Program (TCGA) gene expression data and clinical information and defines the immune signature of OC TME (Shen et al., 2022). Accordingly, the OC patients were classified as non-immune, immune-activated and immune-suppressed subtypes with distinct TME status for each. So, more profound strategies for determining the immune-status of OC patients are still in urgent need to explain the specific molecular mechanisms and guide the development of more effective immunotherapy targets for OC patients.

## 3 cGAS/STING signaling and OC

### 3.1 Overview of cGAS/STING signaling pathway

**FIGURE 3:**
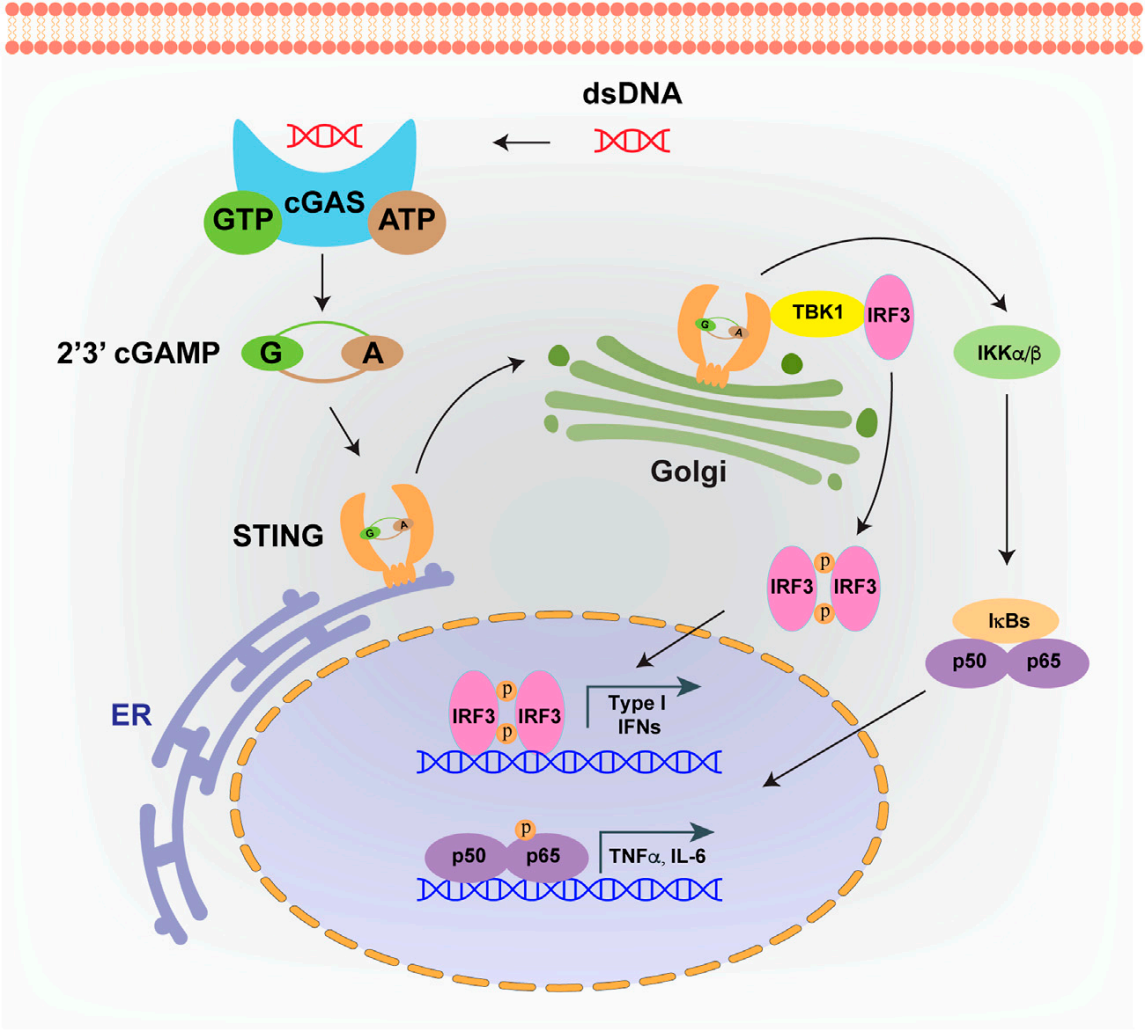
cGAS-STING signaling pathway. Intrinsic or foreign double strand DNA (dsDNA) could be recognized by cGAS. Upon DNA binding, cGAS will catalyze ATP and GTP into 2′,3′-cyclic GMP-AMP (cGAMP). Then cGAMP binds to STING, which facilitates its translocation from endoplasmic reticulum (ER) to Golgi apparatus. cGAMP bound STING on Golgi will recruit TBK1 and IKKs, which leads to the activation of IRF3 and NF-κB, and finally induces the production of type I interferons and inflammatory cytokines.

Both pathogenic and host-derived DNA are immunogenic, which is recognized by an intracellular sensor, named cyclic GMP-AMP synthase (cGAS) (Sun et al., 2013). cGAS binding of double stranded DNA (dsDNA) is sequence-independent, leading to cGAS dimerization and forming a 2:2 complex with DNA, which means each cGAS protein can bind a dsDNA molecule individually (Li et al., 2013). Upon activation, the DNA bound cGAS undergoes conformational changes which are more apt to catalyze ATP and GTP into 2′,3′-cyclic GMP-AMP (cGAMP) (Zhang et al., 2020b). Subsequently, cGAMP is detected by an endoplasmic reticulum (ER) with a localized adaptor protein, called the stimulator of interferon genes (STING) (Cai et al., 2014). After cGAMP recognition, STING translocates to the Golgi apparatus, and subjects to palmitoylation at two cysteine residues (Cys88 and Cys91). The downstream TANK-binding kinase 1 (TBK1) is recruited, and the C-terminus of STING is phosphorylated, which provides a docking site for IRF3 to impel its phosphorylation and activation (Mukai et al., 2016; Ablasser, 2019; Zhang et al., 2019). In addition, transcriptional activity of NF-κB could also be stimulated by STING binding of IκB kinase, and pro-inflammatory signals are switched for cytokines production, including IFNα and IFNβ (Yu and Liu, 2021) (Figure 3). In turn, IFNs are recognized by the heterodimeric receptors IFNAR1/IFNAR2 to activate JAK1, leading to the phosphorylation of members of the signal transducer and activator of transcription (STAT) family and the transcription of IFN stimulating genes (ISGs) (Gan et al., 2021).

STING is the most important adaptor protein facilitating DNA sensing. Since STING mediated signaling activation frequently enhances the ability to combat cancer, and it is restricted within tumor cells, inactivation of STING pathway is prevalent across cancer types (Amouzegar et al., 2021). In OC, the expressional pattern of STING has been characterized across ovarian tumor histotypes. Huvila et al. (2021) reported that STING expressed highly in low grade serous ovarian carcinoma and serous borderline tumors, heterogeneously expressed in high-grade serous and endometrioid carcinomas, but with low levels in clear cell and mucinous carcinomas. Such expression pattern of STING across OC subtypes may suggest differed functions of STING in different cellular origins of OC tumorigenesis. Indeed, cGAS-STING signaling pathway has been revealed as a “double edged sword” in various cancer types, including OC (Du et al., 2022). cGAS-STING pathway mediates tumor immunity for immune surveillance and clearance and seems to exert tumor suppressive effects in cancer related therapies such as radiation therapy, chemotherapy and immunotherapy.

### 3.2 cGAS-STING pathway in OC progression

STING activation is reported to drive carcinogenesis by creating tumor-prone inflammatory conditions or inhibiting T cell mediated anti-tumor immunity effects (Ahn et al., 2014a; Ahn et al., 2014b; Larkin et al., 2017). Ahn et al. reported that STING is required for promoting the inflammatory cytokine levels, and *STING* knockout mice are more resistant to skin carcinogenesis induced by DMBA, which implied that STING induced inflammation is involved in tumor development. Indeed, cumulative evidence implied that sustained activation of cGAS/STING signaling pathway can create an immune suppressive TME that favors tumor progression (Kwon and Bakhoum, 2020). Although the direct link between OC carcinogenesis and STING has not yet been established, Bruand et al. (2021) confirmed a novel role for STING induced immunosuppression dependent on vascular endothelial growth factor A (VEGFA) in ovarian carcinoma cells lacking BRCA1 DNA repair associated (BRCA1). On the contrary, STING has been intensively investigated for its anti-tumor effects, and it is well documented that exogenous stresses induced DNA damage activates the cGAS/STING pathway, thereby causing upregulation of cytotoxic interferons or infiltrating of T lymphocytes. It is demonstrated that targeting STING pathway is effective in inducing antitumor response. Ghaffari et al. (2018) announced that agonists of STING protein could reduce OC induced ascites formation and tumor aggressiveness. Furthermore, Poly (ADP-ribose) polymerase (PARP) inhibition enhances cytosolic DNA fragments accumulation and elicits antitumor responses in mice with BRCA1-deficient OC through STING-dependent pathway (Ding et al., 2018; Shen et al., 2019). In addition, Matthew Knarr et al. (2020) found that miR-181a promoted tumorigenesis in fallopian tube secretory epithelial cells through induction of genomic instability by simultaneously targeting RB1 and STING.

### 3.3 cGAS/STING signaling pathway modulation in OC

To avoid the tumor suppressive effects of cGAS/STING mediated signaling transduction, cancer cells adapt to lower the baseline activity of cGAS-STING signal axis, and thus escape the cGAS mediated innate immune surveillance. In human OC cells, the cGAS-STING pathway related protein expression or activation was modulated by epigenetic or post-translational modifications. Queiroz et al. recently reported that frequent impairment of cGAS-STING signaling pathway in OC cells is largely attributed to the hyper-methylation of the promoter regions for both cGAS and STING genes. Oncolytic viruses have been engineered to selectively amplify and destroy cancer cells without dissolving normal cells, which induce systemic anti-tumor immunity. cGAS-STING pathway in cancer cells could be activated when being infected with engineered vaccinia virus, adenovirus and herpes simplex virus type 1. The ability for DNA virus clearance is blocked in most OC patients and cell lines with defective cGAS-STING pathway, which makes the OC cells are more susceptible to oncolytic virus infection and always show higher sensitivity than normal cells (de Queiroz et al., 2019). Therefore, for cancers loss of cGAS-STING function, oncolytic virus is prospective in combating this immune evasion, and the therapeutic effects can be predicted by measuring expressional levels of cGAS or STING in biopsy specimens. Besides, Zhang et al. reported an alternative way for cGAS-STING signaling attenuation. They discovered that cGAS-STING activation facilitated the binding of USP35 deubiquitinase to STING, which resulted in downregulation of STING poly-ubiquitination and eventually inhibition of type I interferon production (Zhang et al., 2021). Collectively, the data implied that the cGAS-STING signaling pathway could be harnessed to provide a new opportunity to boost immune surveillance of OC.

### 3.4 Combined therapeutic strategies with cGAS/STING signaling pathway for OC

Currently, reduction surgery in combination with platinum/taxane chemotherapy is the standard treatment for high-grade serous ovarian carcinoma (HGSOC). However, more than 70% patients will recur although they firstly respond well to platinum (Ghaffari et al., 2018). IFN stimulating strategies based on STING targeting have gotten attention for cancer treatment and showed optimal therapeutic efficacy in synergy with other therapeutic approaches. Ghaffari et al. (2018) showed that STING agonists combined with anti-PD-1 antibodies greatly promoted IFN response and MHC class II gene expression, and enhanced the therapeutic effect of carboplatin in a mouse model of highly malignant serous ovarian cancer. Grabosch et al. (2019) demonstrated that cisplatin treatment could modulate the immune environment *via* activating the STING pathway, and thus enhances tumor immunogenicity and clearance by increasing adaptive immunity related molecules and T-cell infiltration in mouse EOC models. They also found cisplatin up-regulates PD-L1 expression of OC cells, and when being treated in combination with PD-1/PD-L1 blockade, survival rate of mice bearing aggressive tumors could be significantly increased.

PARP inhibitors (PARPi) have been approved for clinical therapy in OC patients harboring *BRCA1* or *BRCA2* mutation (Ledermann et al., 2012). PARPis are harmful and toxic to OC cells with deficiency in DNA repair *via* specifically inducing the amount of double strand breaks. Shen et al. uncovered that PARPi treatment activated the cGAS/STING pathway and increased tumor infiltrating lymphocytes (TILs), which collectively resulted in an enhanced immunogenic response; the combination treatment of PARPi with immune checkpoint blockade greatly enhanced tumor susceptibility to PD1/PD-L1 administration (Meng et al., 2021). While the cGAS-STING pathway has exhibited prospective as an intervention target in pre-clinical ovarian cancer models, the clinical potential of this strategy needs further exploration.

## 4 Conclusion

As the key innate immune system receptors, TLRs and cGAS-STING are essential in controlling tumor development, progression, and relevant therapeutic outcomes. Expressions of these two kinds of receptors are commonly found in OC cells and TME immune cells. Induced inflammation by dysregulation of TLRs and cGAS-STING pathways has been revealed to be important for OC initiation. However, the pro- or anti-tumor effects for both types of receptors are tumor context dependent, and the mechanisms underlying the pro- or anti-tumor responses require further investigation. In addition, interventions targeting the TLRs and cGAS-STING signaling pathways have been implemented to optimize the efficacy of traditional therapies, especially chemotherapeutic agents for ovarian cancer patients. In order to provide insight into optimizing therapeutic combinations, it is fundamental to decipher precisely how the receptors act through tumor cell-intrinsic pathways and TME cell-mediated immune regulatory pathways. While many of the contemporary preclinical and early-phase clinical studies have conveyed promising results, the duplicitous effects of TLRs and cGAS-STING pathways require in-depth studies to maximize clinical outcomes. For guiding the future clinical treatment strategy in targeting inflammatory in OC patients, the role of cancer cell-induced and microenvironmental immune cell-related inflammation factors should be rigorously discriminated by integrating single cell sequencing and quantification of inflammatory mediators to reveal the abundance of different cell types, the expressional level of the indicated receptors for each cell type, and finally converge to the findings of the most suitable interventional targets.

## Glossary

AO-1: Atractylenolide I
cGAS: cyclic GMP-AMP synthase
CSCs: cancer stem cells
dsRNA: double stranded RNA
DAMPs: damage associated molecular patterns
DCs: dendritic cells
EOC: epithelial ovarian cancer
ER: endoplasmic reticulum
HGSOC: high-grade serous ovarian carcinoma
IL-10R: interleukin-10 receptor
IL12: interleukine 12
ISGs: IFN stimulating genes
IRF3: interferon regulatory factor 3
LRRs: leucine-rich repeats
MDSCs: myeloid-derived suppressive cells
MD-2: myeloid differentiation protein-2
NK: natural killer
OC: ovarian cancer
PRRs: pattern recognition receptors
PAMPs: pathogen associated molecular patterns
P-MAPA: protein aggregate magnesium-ammonium phospholinoleate palmitoleate anhydride
PGN: peptidoglycan
PARP: Poly (ADP-ribose) polymerase
STING: stimulator of interferon genes
STAT: signal transducer and activator of transcription
TLRs: Toll like receptors
TME: tumor microenvironment
TIR: Toll-IL-1 receptor
Treg: regulatory T
TAMs: tumor associated macrophages
TBK1: TANK-binding kinase 1
TILs: tumor infiltrating lymphocytes
